# Prenatal Screening for Common Aneuploidy in Southeast Asian Countries: A Systematic Review of Challenges and Opportunities

**DOI:** 10.1002/pd.70171

**Published:** 2026-05-23

**Authors:** Rapphon Sawaddisan, Lucinda Freeman, Erin Turbitt, Alison McEwen

**Affiliations:** ^1^ Faculty of Health Graduate School of Health Genetic Counselling University of Technology Sydney Sydney Australia; ^2^ Department of Obstetrics and Gynecology, Faculty of Medicine Prince of Songkla University Hat Yai Thailand

## Abstract

Southeast Asian (SEA) countries face persistent challenges in equitable healthcare delivery. Given the high prevalence of aneuploidy, increasing use of advanced prenatal screening technologies, and challenges reported in other regions, this study aimed to identify and assess existing policies and guidelines as well as the enablers and barriers to implementing prenatal aneuploidy screening in SEA. Using the PRISMA framework, we searched seven databases, conducted web searches, reviewed reference lists, and gathered input from key informants. Narrative synthesis was used to interpret and summarise data. We included 83 published documents and verbal insights from four key informants, identifying 17 topics on enablers and barriers grouped into four domains: (1) policies and guidelines, (2) healthcare infrastructure, (3) access to screening, and (4) culture and attitudes of healthcare providers and parents. Effective implementation requires surveillance of aneuploidy and screening practices to understand their impact on public health and national economies. Policy development should consider test performance, healthcare capacity, infrastructure readiness, budget, stakeholder perceptions, cultural norms, and legal frameworks, and be translated into clear guidelines. Sustainable implementation depends on continuous monitoring, ongoing education, collaboration, and regular policy updates.

## Summary

- What is already known about this topic?
  ◦ Southeast Asian countries have introduced prenatal aneuploidy screening while facing various healthcare system challenges.
  ◦ Globally, prenatal aneuploidy screening programs face infrastructure, ethical, and economic barriers, which may also affect their adoption and sustainability in Southeast Asia.
- What does this review add?
  ◦ This review identifies specific enablers and barriers to prenatal aneuploidy screening in Southeast Asia across four domains: (1) policies and guidelines, (2) healthcare infrastructure, (3) access to screening, and (4) culture and attitudes of healthcare providers and parents.
  ◦ This review outlines a practical framework including: surveillance, context‐driven policy development, clear guidelines, education, ongoing monitoring, and stakeholder collaboration.

## 1 Introduction

Southeast Asia (SEA), home to 11 countries and 9% of the global population [1], is characterised by substantial socioeconomic diversity. Based on World Bank classifications, the region includes high‐income economies (Singapore and Brunei Darussalam), upper‐middle‐income countries (Malaysia, Thailand, and Indonesia), and predominantly lower‐middle‐income countries across the remainder of SEA (Cambodia, Lao PDR, Myanmar, Philippines, Timor‐Leste, and Vietnam) [2]. Across this diverse region, persistent challenges remain in delivering equitable, high‐quality healthcare [3, 4, 5, 6]. Regional collaborations through various organisations, including the Association of Southeast Asian Nations (ASEAN) and the Asia Pacific Society of Human Genetics, are aiming to strengthen healthcare systems, including maternal and child health and genetic services [6, 7].

Aneuploidy refers to an atypical number of chromosomes, most commonly presenting as Down syndrome (Trisomy 21), Edwards syndrome (Trisomy 18), or Patau syndrome (Trisomy 13) [8]. Whilst trisomy 18 and trisomy 13 are associated with high early mortality [9], individuals with trisomy 21 often survive but require long‐term medical support [10]. Aneuploidies can substantially affect the quality of life for affected individuals and their families [11], requiring healthcare systems to provide adequate support [12].

Screening during pregnancy enables an early identification of aneuploidy risk, supporting informed reproductive decisions and preparation for care [8, 13]. Current proven methods for aneuploidy screening include ultrasound measurements for nuchal translucency (NT) and nasal bone length (NBL), foetal anatomical scans, maternal serum analytes screening, and non‐invasive prenatal screening tests (NIPT) [8].

Advances in prenatal aneuploidy screening (hereafter, prenatal screening) influence policies, reimbursement, and legal frameworks globally [8, 13, 14]. In SEA, over 6400 cases of Down syndrome were reported in 2019 [15], and most countries have introduced screening programs [16, 17, 18, 19, 20].

Despite global evidence on challenges in implementing prenatal screening, including limited resources [21, 22], protocol violations [16], inequitable access [23], poor‐quality counselling [16, 22], and ethical concerns surrounding pregnancy termination [22, 24], no systematic review has examined these challenges in SEA. A 2024 global review of NIPT programs included only two SEA studies (Thailand and Singapore), both focused on screening and unchanged pregnancy termination rates [25]. To address this gap, we conducted a systematic review of policies, guidelines and research publications to identify the enablers and barriers to implementing prenatal screening in SEA.

## 2 Methods

### 2.1 Study Design

We performed a systematic review following the Preferred Reporting Items for Systematic Reviews and Meta‐Analyses (PRISMA) guidelines, examining policies, guidelines, and published studies related to prenatal screening in SEA countries [26]. The protocol was registered on July 5, 2024 to the International Prospective Register of Systematic Reviews (PROSPERO, CRD42024562066) (link to Prospero protocol https://www.crd.york.ac.uk/PROSPERO/view/CRD42024562066).

The search strategy consisted of (1) database search: we searched Medline, Embase, CINAHL, PsycINFO, MIDIRS, Scopus, and Overton, with guidance from a Health Sciences Librarian to ensure comprehensive coverage, (2) web search: we manually searched the official Ministry of Health and national Obstetrics and Gynaecology Society websites for policies/guidelines. Additionally, we used Google Advanced Search to identify both policy/guidelines and research papers, limiting screening to the first 100 results. Snowballing was used to follow relevant links during the search, (3) reference list review from screened documents, and (4) contacting key informants (physicians, obstetricians, maternal and foetal medicine specialists, and genetic counsellors) via professional networks, and contact emails from each country's Ministry of Health and the main Obstetrics and Gynaecology organisation. Key informants were approached up to three times before data was deemed unavailable.

Database and Google searches used combined search terms related to prenatal screening, common aneuploidies (trisomy 21, 18, and 13), and SEA countries (Supporting Information S1: Appendix I), and were restricted to English‐ and Thai‐language publications. No language restrictions were applied when searching government or professional organisation websites or when identifying national policies and guidelines. Where policy documents were not available in English or Thai, documents in local languages were translated using Google Translate, and documents obtained through key informants were reviewed with the informants prior to manuscript finalisation. The publication period was restricted to 2010 onwards to reflect the introduction of NIPT [27]; when no policies or guidelines published after 2010 were available, the most recent document from each country was reviewed.

### 2.2 Study Criteria

The inclusion and exclusion criteria (Table 1) were formulated using the PICO framework (Population, Intervention, Comparison and Outcome) [28]. We included all available policies/guidelines, publications, and verbal insights from key informants relevant to the enablers and barriers to prenatal screening implementation in SEA countries. Prenatal screening methods include NT, nasal bone measurement, second‐trimester anomaly scans, maternal serum analyte screening, and NIPT.

**TABLE 1:** Study inclusion and exclusion criteria.

Inclusion	Exclusion
Population: We included policies/guidelines and studies whose participants are	
Pregnant women, prospective parents (or families) with nationality in SEA countries.	
Healthcare providers who practise in SEA countries.	
Country‐wide stakeholders: Policymakers, government, general populations, insurance company	
Interventions: Interventions of interest are all the current prenatal common aneuploidy screening methods:	
Foetal first‐ and second‐trimester ultrasound for common aneuploidy screening [*Down syndrome* (*trisomy 21*), *Edward syndrome* (*trisomy 18*) *and Patau syndrome* (*trisomy 13*)], including NT and nasal bone measurement, and routine second‐trimester anomaly scan.	
Maternal serum analytes screening† (1) first‐trimester serum analytes screening (PAPP‐A, hCG, +/− AFP) (2) second‐trimester screening (quadruple screen; hCG, AFP, uE3, DI, or triple screen; hCG, AFP, uE3) (3) combined first‐ and second‐trimester serum analyte screening (PAPP‐A + quadruple screen)	
First‐trimester ultrasound combined with maternal serum analytes screening‡ (1) first‐trimester NT measurement plus serum analytes screening (NT + PAPP‐A, hCG, +/− AFP) (2) first‐trimester NT measurement plus second‐trimester screening (NT + quadruple screen or triple screen) (3) first‐trimester NT measurement plus first‐ and second‐trimester serum analyte screening — Integrated screening — Sequential stepwise screening — Sequential contingent screening	
Any of the † or ‡ plus a routine second‐trimester anomaly scan	
Non‐invasive prenatal testing (NIPT)	
Comparison: No comparison	
Outcomes: We focussed on the following outcomes of interest	
1. Policies and/or guidelines on the use of prenatal common aneuploidy screening in SEA countries.	1. Written policies that do not have a national, provincial, or local health district focus (e.g., statements or practise guidelines for a single institution). 2. Policies are written by patient, family or consumer organisations/support groups, and commercial companies, e.g., leaflets from manufacturer companies. 3. Written policies that are not concerned with prenatal common aneuploidy screening. 4. Scientific studies that are not concerned with prenatal common aneuploidy screening (e.g., screening for solely sex chromosome aneuploidies, other rare genetic diseases or focussing on definitive prenatal diagnosis of any abnormalities).
2. Enablers and barriers to the implementation of prenatal common aneuploidy screening in SEA countries and proposed strategies.	5. Scientific studies that have been developed for commercialisation rather than academic purposes.
3. Accuracy of each screening method when tested in pregnant women in each Southeast Asian country.	
4. The cost‐effectiveness of each prenatal aneuploidy screening method?	
5. Ethical concerns reported by parents, family, healthcare providers, the general population, government and policymakers.	

Abbreviations: AFP, alpha‐fetoprotein; DI, dimeric inhibin A; hCG, human chorionic gonadotropin; NT, nuchal translucency; PAPP‐A, pregnancy‐associated plasma protein A; uE3, unconjugated oestriol.

### 2.3 Study Selection

We used Covidence (Veritas Health Innovation, Melbourne, Australia; www.covidence.org), a web‐based systematic review management tool to screen records. A primary (RS) and a secondary (AM) reviewer independently piloted 10% of titles/abstracts and 20% of full texts. Google search results were logged in Excel, with the first 10% independently screened. Inter‐rater agreement was assessed using a prevalence‐adjusted bias‐adjusted Kappa of ≥ 0.60, indicating moderate agreement [29]. English publications were independently screened, while Thai publications were initially screened and then translated into English using Google Translate, verified by RS and further reviewed by AM. Discrepancies were resolved through joint review and consensus.

### 2.4 Data Extraction and Quality Assessment

Data extracted included study details, population, methodology, intervention, relevant outcomes, and study quality using the Joanna Briggs Institute [30] adapted Excel form. Two reviewers (RS, AM) piloted the initial 10% of data extraction and adjusted the form as needed. The extracted data were compared, and discrepancies were resolved through discussion. Missing data were requested from the authors up to three times. Studies with irretrievable or duplicated data were excluded.

Two reviewers (RS, AM) independently assessed study quality using the QualSyst tool [31] or AGREE II [32]. (Supporting Information S2: Appendix II), resolving disagreements through discussion or a third reviewer.

### 2.5 Narrative Synthesis

We performed a systematic narrative synthesis to interpret, describe, and summarise the variation in prenatal screening across SEA countries and factors influencing implementation success [33].

## 3 Results

**FIGURE 1:**
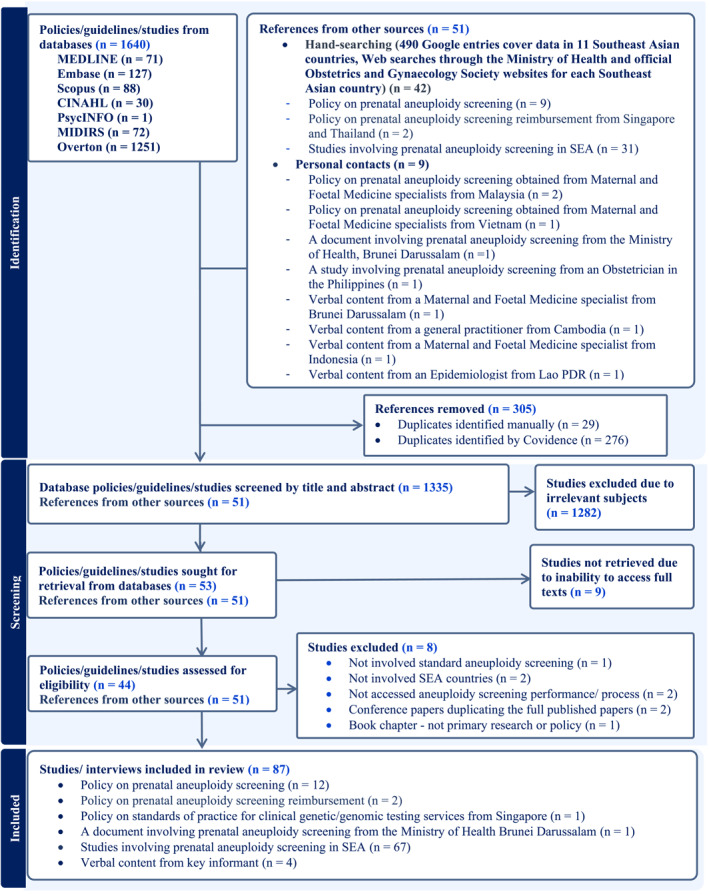
Flow chart demonstrates literature screening and selection as per the PRISMA guidelines.

We identified 1640 potential records through database searches (latest search: 10 December 2025) and retrieved 490 entries through structured Google searches, government websites, and outreach to key informants. After removing duplicates, two reviewers screened 10% of titles/abstracts and 20% of full texts from the databases, achieving Cohen's kappa of 0.76 and 0.75, respectively. RS then screened the remaining records. For the Google search results, 10% screening by two reviewers yielded a kappa of 0.66, after which RS screened the remaining results. In total, 83 documents related to prenatal screening in SEA were included, comprising 15 policies/guidelines, 67 studies, and a document on NIPT from the Ministry of Health, Brunei Darussalam. Verbal data from four key informants from Brunei Darussalam, Cambodia, Indonesia and Lao PDR were included in the study (see Figure 1 for PRISMA flow diagram).

We found evidence that prenatal screening is available in all SEA countries except Timor Leste. In Myanmar, the only available evidence of prenatal screening was an NIPT advertisement on LinkedIn (Pulse and Purpose) and WHO data indicating ultrasound screening for foetal anomalies at selected centres [34].

Table 2 outlines the guidelines, policies, and publications reviewed in this study. Table 3 provides a summary of prenatal screening methods and reimbursement policies in countries with established guidelines on aneuploidy screening or antenatal care that include such screening.

**TABLE 2:** Policies, guidelines and publications reviewed in this study.

Types of documents	Countries (n)	References
Policies/guidelines (*N* = 15)		
Policies/guidelines on prenatal aneuploidy screening (*n* = 7)	Singapore (*n* = 1)	Singapore [35]
	Thailand (*n* = 5)	Thailand [36, 37, 38, 39, 40]
	Vietnam (*n* = 1)	Vietnam [41]
Guidelines on antenatal care and incorporated prenatal aneuploidy screening (*n* = 5)	Malaysia (*n* = 2)	Malaysia [42, 43]
	Thailand (*n* = 2)	Thailand [44, 45]
	The Philippines (*n* = 1)	The Philippines [46]
Policies/regulations about prenatal aneuploidy screening reimbursement (*n* = 2)	Singapore (*n* = 1)	Singapore [47]
	Thailand (*n* = 1)	Thailand [48]
A regulation on standards of practise for clinical genetic/genomic testing services and clinical laboratory genetic/genomic testing services (*n* = 1)	Singapore (*n* = 1)	Singapore [49]
Publications addressing the implementation of prenatal aneuploidy screening within SEA countries (*N* = 67)		
Peer‐reviewed studies that addressed the enablers and barriers to prenatal aneuploidy screening in SEA (*n* = 28)	Malaysia (*n* = 2)	Malaysia [50, 51]
	Singapore (*n* = 4)	Singapore [18, 52, 53, 54]
	Thailand (*n* = 17)	Thailand [55, 57, 58, 59, 60, 61, 62, 63, 64, 65, 66, 67, 68, 69, 70, 71]
	The Philippines (*n* = 2)	The Philippines [19, 72]
	Vietnam (*n* = 2)	Vietnam [73, 74]
	Indonesia, Thailand and Vietnam (*n* = 1)	Indonesia, Thailand and Vietnam [75]
Peer‐reviewed studies that addressed prenatal aneuploidy screening performance in SEA (*n* = 29)	Singapore (*n* = 2)	Singapore [76, 77]
	Thailand (*n* = 22)	Thailand [60, 61, 62, 63, 64, 65, 66, 67, 78, 79, 80, 81, 82, 83, 84, 85, 86, 87, 88, 89, 90, 91]
	Vietnam (*n* = 4)	Vietnam [17, 92, 93, 94]
	Indonesia, Thailand, Vietnam (*n* = 1)	Indonesia, Thailand, Vietnam [75]
Peer‐reviewed studies that addressed the cost‐effectiveness of prenatal aneuploidy screening in SEA (*n* = 6)	Thailand (*n* = 5)	Thailand [56, 57, 95, 96, 97]
	Vietnam (*n* = 1)	Vietnam [98]
Genetic counselling thesis addressing the enablers and barriers to prenatal aneuploidy screening in the Philippines (*n* = 1)	The Philippines (*n* = 1)	The Philippines [99]
Editorials on prenatal aneuploidy screening (*n* = 11)	Indonesia (*n* = 2)	Indonesia [100, 101]
	Indonesia, the Philippines, Malaysia, Singapore, and Thailand (*n* = 1)	Indonesia, the Philippines, Malaysia, Singapore, and Thailand [102]
	The Philippines (*n* = 8)	The Philippines [103, 104, 105, 106, 107, 108, 109, 110]
A report from the workshop at the 10th Asia pacific conference on human genetics (*n* = 1)	Indonesia, Malaysia, the Philippines, Thailand (*n* = 1)	Indonesia, Malaysia, the Philippines, Thailand [111]
Thailand pilot study for the prevention of down syndrome (*n* = 1)	Thailand (*n* = 1)	Thailand [16]
World health organisation, regional office for South‐East Asia report (*n* = 1)	Myanmar (*n* = 1)	Myanmar [34]
Documentation on NIPT, provided by the ministry of health Brunei Darussalam (*N* = 1)	Brunei Darussalam (*n* = 1)	Brunei Darussalam (Ministry of health, Brunei Darussalam: Email correspondence, 29 Oct 2024)

**TABLE 3:** Prenatal aneuploidy screening methods and reimbursement policies in countries with prenatal screening policies or guidelines (Malaysia, Singapore, Thailand, and Vietnam).

Prenatal aneuploidy screening methods (universally offered in all listed countries)	Singapore (high‐income)	Malaysia (upper‐middle‐ income)	Thailand (upper‐middle‐income)	Vietnam (lower‐middle‐income)
Nuchal translucency (NT) measurement	✓ [35]	✓ [42]	✓ [36, 38, 40, 44, 45, 73, 111]	✓ [41]
First‐trimester maternal serum analyte screening		✓ [42]	✓ [36, 39, 44, 45]	✓ [41]
Combine first‐trimester screening (NT + first‐trimester maternal serum analyte)	✓ [35]	✓ [42]	✓ [36, 38, 39, 40, 44, 45]	✓ [41]
NT combined with second‐trimester maternal serum analyte screening	✓ [35]			
Second‐trimester maternal serum analyte screening	✓ [35]	✓ [42]	✓ [36, 38, 39, 40, 44, 45]	✓ [41]
Stepwise sequential screening test (combined first‐trimester screening followed by second‐trimester screening or diagnostic testing)	✓ [35]			
Serum integrated test (first‐trimester maternal serum analyte screening followed by second‐trimester maternal serum analyte screening)			✓ [36, 39]	
Fully integrated test (combined first‐trimester screening followed by second‐trimester maternal serum analyte screening)			✓ [36, 39]	
Non‐invasive prenatal testing (NIPT)	Guideline predates NIPT (published 2008)	✓ [42]	✓ [40, 44, 45]	✓ [41]
Second‐trimester ultrasound	✓ [35, 45]	✓ [42, 43]	✓ [37, 40, 44, 45]	✓ [41]
Reimbursement policies	Partially reimbursed prenatal aneuploidy screening under medisave as pre‐delivery medical expenses [47]		The national health Security office reimburses second‐trimester serum screening (quadruple test), NIPT for common aneuploidies, follow‐up diagnostic procedures, and pregnancy termination for confirmed genetic abnormalities (if elected), and provides a 400‐baht subsidy for routine antenatal ultrasound examinations [48]	

Barriers and enablers of prenatal screening implementation in SEA were categorised into domains.

### 3.1 Domain 1: Policies and/or Guidelines

#### 3.1.1 Enablers

##### 3.1.1.1 Comprehensive Antenatal Care in a Single Guideline

Comprehensive guidelines integrating prenatal screening with antenatal care aspects support healthcare providers (HPs) in delivering standardised practice [16]. Examples include two Thai guidelines and one Malaysian guideline that provide trimester‐specific screening recommendations throughout antenatal care [42, 44, 45].

#### 3.1.2 Barriers

##### 3.1.2.1 No National Policies/Guidelines

Prenatal screening is evidently available in 10 out of 11 SEA countries; however, only Malaysia, Singapore, Thailand and Vietnam have national guidelines. In the Philippines, the lack of guidelines limits the provision of NIPT [72], while Singapore reports inconsistencies in NIPT use and calls for formal guidance [52].

##### 3.1.2.2 Guidelines Contain Areas for Improvement

A training guideline for Thai HPs shows minor inconsistencies in risk factors, gestational age for confirmatory tests, and detection rates. The section on aneuploidy screening methods does not include NIPT, despite its availability in Thailand at the time of publication (2020) [36].

### 3.2 Domain 2: Healthcare Infrastructures

#### 3.2.1 Enablers

##### 3.2.1.1 Formal and Ongoing Education for HPs

Formal genetics education has been available since the 1990s and has been integrated into undergraduate medical school curricula in Indonesia, the Philippines, Malaysia, Singapore, and Thailand [102]. Initially, specialists received training abroad in countries such as the United States and Australia with the government's financial support (e.g., Malaysia and the Philippines) [102, 104]. Genetic counselling education began in Indonesia, the Philippines, and Malaysia between 2006 and 2015 [102, 105]. Singapore lacks a local genetic counselling programme, but services are provided by overseas‐trained HPs [102].

Continuing genetics and genetic counselling education is evident across the region, including workshops in Malaysia and Thailand [111], government‐led training during Thailand's maternal serum screening reimbursement pilot [16], and on‐the‐job training in Vietnam's prenatal centres [73].

Regional genetics organisations, such as the Asia Pacific Society of Human Genetics (APSHG) and the Professional Society of Genetic Counsellors in Asia (PSGCA) are actively providing ongoing education for HPs through courses, webinars and meetings [102, 106].

##### 3.2.1.2 Government Regulation and Evaluation of Prenatal Screening Processes

Singapore and Thailand literature highlighted the need for government regulation to ensure standardised prenatal screening and appropriate reimbursement [16, 35, 49, 56, 57].

A 2016 Thai pilot study on maternal serum screening highlighted improvements needed in lab procurement, counselling, data collection, specimen handling, and evaluation system usability [16].

Cost‐effectiveness studies in Thailand informed reimbursement policy [56, 57, 95, 96, 97], showing that although NIPT is more expensive, it can be the most cost‐efficient due to high detection rates and fewer false positives, reducing invasive procedures, associated risks, and the need for specialised staff and laboratories [56, 57, 96]. In early 2025, Thailand approved reimbursement for universal NIPT in addition to the existing coverage for the Quadruple test [48].

##### 3.2.1.3 Collaboration Among HPs, Parents and Policymakers

Regional and international collaborations among HPs, such as Indonesia‐Netherlands, Malaysia‐Australia [102], and the Philippines‐United States [104], have supported genetic education and capacity building [102, 104, 105, 106]. HP input contributed to context‐specific policy development in Indonesia and Thailand [16, 100], while joint efforts of HPs, and parents in the Philippines strengthened genetic services through knowledge exchange and advocacy for improved governmental support [102].

#### 3.2.2 Barriers

##### 3.2.2.1 Inadequate Personnel, Laboratories, Equipment and Economic Challenges

Cambodia, Indonesia, Lao PDR, the Philippines, Thailand, and Vietnam face economic and infrastructure challenges. (Ho C, Personal contact, October 1, 2024; Aryananda R. Personal contact, October 9, 2024; Soukkhaphone B, Personal contact, September 26, 2024) [16, 44, 107, 111].

NT measurement is largely unavailable in primary healthcare settings in Indonesia, and Cambodia due to shortages of trained personnel. Both countries lack sufficient government laboratories, and maternal serum analyte screening is limited to select hospitals partnering with private laboratories. (Aryananda R., personal contact, October 9, 2024; Ho C., personal contact, October 1, 2024).

The limited laboratory availability in Thailand forced hospitals to send serum samples for long distances [55]. A 2012 Thai study found that transport delays combined with high ambient temperatures, were associated with increased Quadruple test false‐positives [55].

Vietnam experiences shortages of trained personnel and equipment for confirmatory diagnostics [73], and Malaysian public hospitals often lack routine anomaly scans, leading to late diagnoses and increased maternal risk [50]. Workforce shortages in the Philippines and Thailand reduce patient interaction and compromise genetic counselling [16, 44, 105], whilst inadequate infrastructure in the Philippines further limits specialist training opportunities [107].

In 2016, fiscal constraints delayed Thailand's pilot maternal serum screening as some provinces could not start due to budget limits [16].

##### 3.2.2.2 Inadequate Knowledge and Training for HPs

Some HPs in the Philippines, Thailand, Singapore, and Vietnam had inadequate knowledge regarding prenatal screening and counselling [16, 52, 72, 73, 99]. Some in the Philippines and Singapore were unaware that NIPT is a screening test, and the results have to be confirmed [52, 72]. Some Filipino HPs lack knowledge about the follow‐up services, such as genetic counselling, ultrasound, and amniocentesis [99], and reported feeling unconfident in offering the screening [72]. In Singapore, HPs face challenges in counselling for complex situations such as NIPT failures and incidental findings [52].

In Thailand, some HPs avoided offering screening due to insufficient skills, mishandled serum samples, entered data inaccurately in serum screening software, and misused terms such as ‘normal/abnormal’ instead of ‘high risk/low risk’ during counselling [16].

Reports from Singapore and Vietnam call for more training in prenatal screening and counselling [52, 73].

##### 3.2.2.3 Inadequate Societal Awareness and Education for Parents

Lower maternal education levels were associated with reduced antenatal care attendance in Indonesia [101]. In Thailand, limited parental understanding of prenatal screening makes counselling and the management of high‐risk results challenging, particularly in time‐constrained settings [16]. Even after counselling, parental understanding could still be inadequate, as reported in Singapore and Thailand, where misconceptions about high‐risk screening results persist [16, 53, 58]. Parent education materials may also be inadequate. A 2015 Thai study found that some parent leaflets lacked key details such as false‐negative rates, reasons for false results, and some incorrectly implied that screening could diagnose Down syndrome [59].

##### 3.2.2.4 Inadequate Surveillance and Research Limit Policymakers' Awareness

There is underreporting of genetic diseases in Indonesia [100], limited data on birth defect cases, prenatal screening rates and genetic counselling quality in the Philippines [99, 105, 108] and Vietnam [73]. Underreporting reduces public awareness of the importance of prenatal screening [73, 99, 105, 108].

Research across SEA remains limited. In Indonesia, national research prioritises infectious and non‐communicable diseases over genetics [100]. Within the available literature, a substantial proportion of screening performance studies originate from Thailand [60, 61, 62, 63, 64, 65, 66, 67, 75, 78, 79, 80, 81, 82, 83, 84, 85, 86, 87, 88, 89, 90, 91]. Additional performance studies have also been reported from Indonesia [75], Singapore [76, 77], and Vietnam [17, 75, 92, 93, 94], reporting high screening accuracy (see Supporting Information S3: Appendix III).

Research validating Western algorithms and defining reference ranges and risk cut‐offs for maternal serum analyte screening is largely conducted in Thailand. [60, 61, 62, 63, 64, 65, 66, 67, 68, 69, 70, 75, 91], with one study from Indonesia [75] and Vietnam [75]. Cost‐effectiveness studies are also limited, reported only from Thailand [56, 57, 95, 96, 97] and Vietnam [98].

### 3.3 Domain 3: Access to Prenatal Screening

#### 3.3.1 Enablers

##### 3.3.1.1 Government Reimbursement of Prenatal Screening

Evidence from Singapore and Thailand suggests that prenatal screening uptake is influenced by cost [16, 18], with government funding in Thailand associated with improved access [16].

Amongst 11 SEA countries, Brunei, Singapore and Thailand reimburse prenatal screening. Brunei offers free NT, second‐trimester ultrasound, maternal serum analytes, and NIPT for high‐risk women and free second‐trimester ultrasound for low‐risk Bruneian nationals. (Ministry of Health of Brunei Darussalam, via email correspondence, October 29, 2024; Rahman RHA, personal contact, September 29, 2025). Singapore provides partial reimbursement through the MediSave Maternity Package [47], Thailand reimburses the Quadruple test (since 2019) and NIPT (since 2025), including follow‐up procedures and pregnancy termination [48]. Through successful negotiations with NIPT providers, the government reduced the cost of NIPT to approximately $76/person [48].

#### 3.3.2 Barriers

##### 3.3.2.1 High Cost of Prenatal Screening

Parents across Cambodia, Indonesia, Lao PDR, the Philippines, Malaysia, Singapore, Thailand, and Vietnam face economic barriers to prenatal screening. In Cambodia, maternal serum screening costs about $64, and NIPT samples sent abroad raise costs to $590–$620. (Ho C, Personal contact, October 1, 2024) In Lao PDR, private hospitals provide all screening, with NIPT samples also sent overseas, costing between $515 and $787. (Soukkhaphone B, Personal contact, September 26, 2024) In Indonesia, NIPT is typically accessed only by parents in private facilities who can afford the $250 fee. (Aryananda R, personal contact, October 9, 2024) In Singapore, some prospective parents were less willing to pay for screening tests that offered higher accuracy [18]. Many Filipino parents cannot afford NIPT, which costs $491–$1103 [19]. Some Vietnamese parents declined further testing after a high‐/moderate‐risk result due to cost [74].

HPs from Malaysia [51], Thailand [71], and Vietnam [73] identify high screening costs as a major barrier, and some Filipino obstetricians avoid offering screening because it is too expensive [72, 99].

##### 3.3.2.2 Geographical Challenges

As archipelagic nations, Indonesia (over 13,000 islands) [100] and the Philippines (over 7000 islands) [105] face significant challenges in ensuring equitable access to antenatal care across regions. In Indonesia, antenatal care utilisation varies widely, and access to healthcare is tied to the country's transportation infrastructure [101]. In the Philippines, prenatal screening services are predominantly available in urban centres [105].

### 3.4 Domain 4: Cultures and Attitudes of HPs and Parents

#### 3.4.1 Enablers

##### 3.4.1.1 Education for Prospective Parents

Researchers from Singapore [18, 53, 54], the Philippines [99], and Filipino parents [19] stressed the importance of providing prospective parents sufficient information to make informed choices. Counselling quality can be enhanced when parents are well‐informed, as observed in the Philippines [99], and through the use of an animated educational video in Thailand [58]. The use of an animated educational video on NIPT alongside counselling was reported to improve parental knowledge and is now part of routine practise at a Thai university hospital [58].

##### 3.4.1.2 Respecting Parental Autonomy and Diverse Cultures

Studies from Singapore show differences in prenatal screening preferences between parents and healthcare providers [18, 53, 54] and parents may perceive risk differently from medical definitions [53], making comprehensive information and respect for autonomy essential [53].

Perceptions of autonomy are shaped by cultural context. In the Philippines, family cohesion strongly influences decision‐making [109]. Evidence from the Philippines and Thailand indicates that cultural beliefs influence how individuals perceive genetic conditions [16, 103, 110]. Counselling practices should adapt to multicultural and multilingual contexts, including family involvement where appropriate [103, 109, 110, 111]. In the Philippines, the recruitment of students from diverse regions to improve access to genetic services for minority groups has been reported [110].

#### 3.4.2 Barriers

##### 3.4.2.1 Attitudes on Prenatal Screening and Parental Autonomy

In the Philippines, some obstetricians view the screening process as overly complex and believe it should not be offered universally. Instead, they suggest it should be based on parental socioeconomic status and willingness for pregnancy termination [99]. In Thailand, a study found that obstetric residents were less supportive of offering universal prenatal screening [112].

##### 3.4.2.2 Strict Beliefs and Laws on Pregnancy Termination

Views on prenatal screening in the Philippines are connected to perspectives on pregnancy termination [19, 99, 107]. Due to strict moral beliefs and the fact that pregnancy termination is considered a criminal act in the country [107], some Filipino HPs choose not to offer screening [99]. Additionally, some parents see little benefit in early diagnosis of Down syndrome, believing it would not influence pregnancy management [19].

To support the interpretation of findings related to pregnancy termination, a contextual table summarising the legal status and gestational limits across SEA is provided (Table 4). This information was not derived from a systematic search.

**TABLE 4:** Legal status of pregnancy termination and gestational age limits in Southeast Asian countries.

Legal status of pregnancy termination	Country	Permitted indications (summary)	Gestational age limit	References
Prohibited	Philippines	No legally permitted indications	—	[113]
Highly restricted	Brunei Darussalam	To save the woman's life	No explicit limit stated	[114]
	Myanmar	To save the woman's life	No explicit limit stated	[115]
Restricted	Indonesia	Maternal or foetal health indications; pregnancy resulting from rape	≤ 14 weeks for rape indication; no clear limit for other indications	[116]
	Lao PDR	Maternal or foetal health indications; pregnancy resulting from rape; selected social and clinical circumstances (case‐by‐case assessment)	No explicit limit stated	[117]
	Malaysia	Maternal health indications	No explicit limit stated	[118]
	Timor‐Leste	Maternal or foetal health indications	No explicit limit stated	[119]
Permitted	Cambodia	On request; maternal or foetal health indications; pregnancy resulting from rape	≤ 12 weeks on request; > 12 weeks based on other indications	[120]
	Singapore	On request; maternal health indications	≤ 24 weeks on request; > 24 weeks for maternal health indications	[121]
	Thailand	On request; maternal or foetal health indications; pregnancy resulting from rape	≤ 12 weeks on request; ≤ 20 weeks for other indications	[122]
	Vietnam	On request	No explicit limit stated	[123]

Table 5 presents the domains, detailing the key enablers and barriers along with supporting references.

**TABLE 5:** Reported enablers and barriers to implementing prenatal aneuploidy screening in Southeast Asia (SEA) by domain, country, and supporting references.

Enablers	Barriers	High‐income countries		Upper‐middle‐income countries			Lower‐middle‐income countries						Overall SEA (not specified)
		Brunei Darussalam	Singapore	Indonesia	Malaysia	Thailand	Cambodia	Lao PDR	Myanmar	Philippines	Timor leste	Vietnam	
Domain: National policies and/or guidelines													
Integration of prenatal care with aneuploidy screening, with defined timeframes for testing.						✓ [16]							
	Absence of national policies and/or guidelines for prenatal aneuploidy screening		✓ [52] (No specific NIPT guidance within existing prenatal screening policies)							✓ [72]			
Domains: Healthcare infrastructure													
Government funding for overseas genetics education					✓ [102]					✓ [104]			
Formal genetic education for HPs			✓ [102]	✓ [102]	✓ [102]	✓ [102]				✓ [102, 105]			
Continuing genetic education for HPs				✓ [111]	✓ [111]	✓ [16, 111]				✓ [111]		✓ [73]	✓ [102, 106]
Supplementary educational tools for prospective parents						✓ [58]							
Collaboration amongst healthcare providers										✓ [104, 105]			✓ [106]
Collaboration between healthcare professionals and the government				✓ [100]		✓ [16]							
Collaborations amongst healthcare providers and parents										✓ [19]			✓ [102]
Government oversight of the implementation of prenatal aneuploidy screening						✓ [16]							
Policy updates via cost‐efficiency reviews						✓ [56, 57]							
Validation of Western non‐biochemical screening models						✓ [67]							
	Fiscal constraints			✓ (Aryananda R., personal contact, October 9)		✓ [16]	✓ (Ho C, personal contact, October 1, 2024)	✓ (Soukkhaphone B, personal contact, September 26, 2024)					
	Birth defect cases and prenatal screening rates are underreported			✓ [100]						✓ [99, 105, 108]		✓ [73]	
	Shortage of prenatal aneuploidy screening service/laboratory			✓ (Aryananda R., personal contact, October 9)	✓ [50]	✓ [55]	✓ (Ho C, personal contact, October 1, 2024)	✓ (Soukkhaphone B, personal contact, September 26, 2024)		✓ [105, 107]		✓ [73]	
	Poor sample handling and transportation					✓ [16, 55]							
	Inadequate genetic counselling service									✓ [99]			
	Shortage of trained staff			✓ [111]	✓ [111]	✓ [16, 44, 111]				✓ [105, 111]		✓ [73]	
	Limited knowledge of healthcare providers in prenatal aneuploidy screening and genetic counselling		✓ [52]			✓ [16]				✓ [72, 99]		✓ [73]	
	Limited genetic training and clinical placements									✓ [107]			
	Absence of ethnic‐specific serum analyte reference ranges affecting screening accuracy			✓ [75]		✓ [60, 62, 63, 64, 65, 66, 68, 69, 70, 75]						✓ [75]	
	Misleading or incomplete informational materials provided to parents					✓ [59]							
	Inadequate societal awareness and parental education		✓ [53]	✓ [101]		✓ [16, 58]							
Domains: Access to prenatal aneuploidy screening													
Reimbursement for prenatal aneuploidy screening						✓ [16]							
	Geographical challenge			✓ [100, 101]						✓ [105]			
	High cost of the screening test		✓ [18]	✓ (Aryananda R, personal contact, October 9, 2024)	✓ [51]	✓ [61, 71]	✓ (Ho C, personal contact, October 1, 2024)	✓ (Soukkhaphone B, personal contact, September 26, 2024)		✓ [19, 72, 99]		✓ [73, 74]	
	Prenatal aneuploidy screening is not reimbursed									✓ [105, 107]			
Domains: Cultures and attitudes of healthcare providers and parents													
Informed parents motivating healthcare provider skill development										✓ [99]			
Sufficient information for prospective parents			✓ [18, 54]							✓ [19]			
Respecting parental autonomy			✓ [53]										
Respecting cultural diversity and integrating multicultural contexts into practise				✓ [111]	✓ [111]	✓ [111]				✓ [109, 111]			
Recruiting students from diverse regions										✓ [110]			
	Healthcare providers' negative attitudes towards prenatal aneuploidy screening					✓ [112]				✓ [99]			
	Cultural diversity			✓ [111]	✓ [111]	✓ [111]				✓ [103, 110, 111]			
	Negative social beliefs about invasive diagnostic procedures for foetal aneuploidy					✓ [16]							
	Cultural and legal views on pregnancy termination									✓ [19, 99, 107]			

## 4 Discussion

We examined enablers and barriers to implementing prenatal screening across 11 SEA countries, finding services available in all except Timor‐Leste. Seventeen topics were grouped into four domains: (1) policies and/or guidelines, (2) healthcare infrastructure, (3) access to prenatal screening, and (4) cultures and attitudes of HPs and parents. Shared challenges across SEA suggest that successful strategies in one country may be adaptable to others.

Most SEA countries are classified as lower‐ or upper‐middle income [2]. Health financing in SEA is constrained by low public funding [124, 125, 126]. Competing priorities, including climate change, nutrition, infectious diseases, and high maternal and child mortality [124], further limit investment in infrastructure and antenatal care research [100, 125, 126, 127].

Limited facilities and workforce capacity were commonly reported in lower‐ and upper‐middle‐income countries [16, 44, 99, 105, 107] (Ho C, Personal contact, October 1, 2024; Aryananda R. Personal contact, October 9, 2024; Soukkhaphone B, Personal contact, September 26, 2024), while high‐income countries like Singapore highlight the need for ongoing education for HPs [52]. Beyond infrastructure, success depends on education systems, transport and telecommunication capacity [100, 101].

Surveillance and research gaps persist, limiting policymakers' ability to allocate resources effectively. Tracking birth defects, monitoring screening programs, and conducting cost‐effectiveness studies are essential for informed policy [73, 99, 100, 105, 108].

Cultural diversity and variation in perceptions of prenatal screening have been reported among prospective parents and HPs in Indonesia [111], the Philippines [19, 99, 103, 107, 109, 110, 111], Malaysia [111], Thailand [16, 111, 112] and Singapore [53]. Communication styles, family cohesiveness, and views on autonomy in SEA can differ from those in Western norms [109]. Spiritual beliefs may complicate counselling and treatment‐seeking [103, 110]. Clinical practice should therefore be responsive to the sociocultural context [109, 111]. When misunderstandings arise about genetic conditions, culturally appropriate genetic education may help mitigate these challenges [109].

Prenatal screening can improve maternal and neonatal outcomes and support family well‐being [8, 13]. Some parents choose prenatal screening to prepare for the birth of a child and opt to continue the pregnancy after a diagnosis is confirmed [128]. Overemphasising pregnancy termination as the main outcome can hinder implementation, as HPs may avoid offering screening, assuming patients would decline pregnancy termination [19, 99]; alternatively, patients may feel pressured to terminate the pregnancy following a confirmed aneuploidy, especially in a social context where disability is viewed negatively [129]. Understanding the broader benefits of prenatal screening is essential to uphold patient autonomy while respecting local legal and ethical frameworks.

Although prenatal screening services are available in most countries, only four have established national policies/guidelines [35, 36, 37, 38, 39, 40, 41, 42, 43, 44, 45, 46]. Developing and regularly updating national guidelines aligned with each country's context can help HPs overcome challenges such as low confidence and limited knowledge and promote consistent practice [16, 35, 49, 52, 56, 57, 111].

Educational gaps among HPs extend beyond clinical knowledge and include an understanding of healthcare pathways [99]. Training should cover practical aspects such as referral processes, cost, and reimbursement policies. The National Society of Genetic Counselors (United States) advises considering state regulations, insurance coverage, and the availability of laboratories when offering prenatal screening [130].

Geographical barriers and limited services affect archipelagic countries such as Indonesia [100, 101, 111] and the Philippines [99, 105, 107, 111], and also in Malaysia [111], Thailand [16, 44, 111], and Vietnam [73]. Telehealth improves access in remote areas in the Philippines [131]. In Australia, telephone and web‐based counselling improved understanding among low socio‐economic populations [132], while a Dutch study demonstrated that a web‐based multimedia decision aid supported informed autonomous choices [133]. These technologies also foster genetic education and cross‐regional collaboration [102, 106]. However, effective use of such technologies requires adequate telecommunication infrastructure and digital literacy among users. In the Philippines, financial barriers limit telehealth services as some parents lack internet access or devices [131].

Thailand illustrates how screening programs can evolve through continuous evaluation. A 2012 study identified specimen transport issues increasing false‐positive rates, prompting improvements in handling and transport for second‐trimester serum screening [55]. The insights on the enablers and barriers from the pilot study in 2016 on maternal serum analyte screening led to enhanced genetic education for HPs and parents, consistent government oversight, reimbursement for prenatal screening for all women, and a better understanding of cultural influence [16]. With cost‐effectiveness research on different screening methods [56, 57, 95, 96, 97], Thailand introduced universal reimbursement for NIPT from 2025 [48].

Barriers commonly reported in SEA, such as limited expertise, inadequate training and guidelines, geographic inequities, and insufficient patient counselling, are also observed in other regions [20, 23]. Globally, countries have adopted varied approaches, shaped by each country's healthcare system and cultural contexts. For example, India restricts disclosure of foetal sex through NIPT to cases with suspected genetic abnormalities due to concerns about sex‐selection. In China, partial reimbursement for NIPT is available in some cities, while in the United Kingdom and Hong Kong offer second‐tier NIPT free of charge to high‐risk women [20].

Policy development should consider country‐specific factors, including test performance, healthcare capacity, infrastructure readiness, stakeholder perceptions, cultural norms, and legal and ethical considerations. Consistent with our findings, the Netherlands, where prenatal screening has been implemented, has identified key enablers, including continuous staff training, standardised counselling, post‐implementation evaluation, and stakeholder collaboration [134].

Our study's strength lies in its comprehensive approach, incorporating data from scientific databases, national policies/guidelines, local publications, and key informant insights. We identified diverse enablers and barriers across medical, economic, and cultural domains. Limitations include potential underreporting in countries with weak surveillance systems and heterogeneity in laboratory methods, which precluded meta‐analysis; consequently, a narrative synthesis was undertaken. Given the limited volume of research in the region, no studies were excluded based on quality assessment; however, all published studies were found to be of acceptable quality and provide scientific merit (Supporting Information S2: Appendix II).

## 5 Conclusion

Effective implementation of prenatal screening in SEA requires robust surveillance to assess the clinical and economic impact. Policies and guidelines should incorporate test performance, healthcare and infrastructure capacity, stakeholder views, and cultural and legal frameworks. Training must cover genetics, counselling, and system workflows. Sustained implementation depends on continuous monitoring, policy updates, education, and collaboration at national and international levels.

## Funding

The authors have nothing to report.

## Ethics Statement

The authors have nothing to report.

## Consent

The authors have nothing to report.

## Conflicts of Interest

The authors declare no conflicts of interest.

## Supporting information


Supporting Information S1

Supporting Information S2

Supporting Information S3

## Data Availability

The authors have nothing to report.
