# Supplementary material for: Prenatal Screening for Common Aneuploidy in Southeast Asian Countries: A Systematic Review of Challenges and Opportunities
Source: Prenat Diagn. 2026 May 23;46(9):1393–410. doi: 10.1002/pd.70171 (PMC13446533; doi:10.1002/pd.70171)
Supplement: Supplementary file 1 [file PD-46-1393-s002.docx]

**Appendix I: Database and web search strategy and keywords**

**1. Example of Medline literature searching: 71 results**

1	Down Syndrome/	27699

2	trisomy 21.mp.	7650

3	down's syndrome.mp.	8301

4	mongolism.mp.	1605

5	trisomy G.mp.	42

6	chromosome 21 duplication.mp.	3

7	chromosome 21 trisomy syndrome.mp.	0

8	Trisomy 18 Syndrome/	710

9	edwards syndrome.mp.	349

10	edward syndrome.mp.	39

11	trisomy 18.mp.	3057

12	trisomy e.mp.	28

13	chromosome 18 duplication.mp.	1

14	chromosome 18 trisomy syndrome.mp.	1

15	Trisomy 13 Syndrome/	615

16	trisomy 13.mp.	2135

17	patau syndrome.mp.	228

18	patau's syndrome.mp.	72

19	chromosome 13 duplication.mp.	0

20	chromosome 13 trisomy syndrome.mp.	1

21	down syndrome.mp.	33840

22	trisomy 18 syndrome.mp.	805

23	trisomy 13 syndrome.mp.	688

24	Abnormal Karyotype/	633

25	abnormal karyotyp*.mp.	3200

26	Aneuploidy/	14721

27	aneuploid*.mp.	30251

28	Trisomy/	12933

29	trisom*.mp.	26533

30	Chromosome Disorders/	20708

31	Chromosom* Disorder*.mp.	22904

32	chromosom* abnormalit*.mp.	21031

33	Chromosome Aberrations/	64432

34	chromosom* aberration*.mp.	78735

35	Chromosome Duplication/	1237

36	chromosom* duplication*.mp.	1925

37	autosom* aberration*.mp.	56

38	Prenatal Diagnosis/	42046

39	prenatal diagnos*.mp.	57742

40	prenatal screening.mp.	4357

41	antenatal screening.mp.	1670

42	antenatal diagnos*.mp.	3082

43	fetal screening.mp.	139

44	fetal diagnos*.mp.	1040

45	foetal screening.mp.	7

46	foetal diagnoses.mp.	1

47	foetal diagnosis.mp.	36

48	intrauterine diagnos*.mp.	222

49	diagnosis, antenatal.mp.	28

50	diagnoses, antenatal.mp.	1

51	diagnosis, intrauterine.mp.	21

52	diagnoses, intrauterine.mp.	1

53	diagnosis, prenatal.mp.	166

54	diagnoses, prenatal.mp.	13

55	Maternal Serum Screening Tests/	580

56	maternal serum screening.mp.	1261

57	maternal serum triple test.mp.	6

58	maternal serum quadruple test.mp.	3

59	Noninvasive Prenatal Testing/	763

60	Noninvasive Prenatal Testing.mp.	1427

61	Non-invasive Prenatal Testing.mp.	1262

62	Noninvasive Prenatal Diagnosis.mp.	417

63	Noninvasive Prenatal Diagnoses.mp.	2

64	Non-invasive Prenatal Diagnosis.mp.	460

65	Non-invasive Prenatal Diagnoses.mp.	4

66	Noninvasive Prenatal Screening.mp.	282

67	Non-invasive Prenatal Screening.mp.	239

68	prenatal cell-free DNA screening.mp.	53

69	prenatal cfDNA screening.mp.	35

70	prenatal cell-free DNA testing.mp.	6

71	prenatal cfDNA testing.mp.	4

72	Nuchal Translucency Measurement/	1560

73	Nuchal Translucency.mp.	3162

74	Ultrasonography, Prenatal/	38399

75	Ultrasonograph*, Prenatal.mp.	38446

76	prenatal ultrasound*.mp.	4093

77	prenatal ultrasonograph*.mp.	1367

78	fetal ultrasound*.mp.	1806

79	fetal ultrasonograph*.mp.	546

80	antepartum ultrasound*.mp.	19

81	antepartum ultrasonograph*.mp.	14

82	fetal echograph*.mp.	34

83	Asia, Southeastern/	9095

84	Asia*, Southeast*.mp.	9296

85	Borneo/	974

86	Borneo.mp.	2191

87	Brunei/	328

88	Brunei.mp.	784

89	Cambodia/	4144

90	Indochina/	51

91	Indochina.mp.	719

92	Indonesia/	16207

93	Laos/	2452

94	Malaysia/	19975

95	Mekong Valley/	36

96	Mekong Valley.mp.	39

97	Myanmar/	3525

98	Myanmar.mp.	6560

99	burma.mp.	1220

100	Philippines/	10488

101	Philippines.mp.	16955

102	Singapore/	17103

103	Thailand/	34007

104	Timor-Leste/	309

105	Timor*.mp.	1324

106	Vietnam/	16764

107	viet nam.mp.	1532

108	southeast* asia*.mp.	26052

109	SEA.mp.	125506

110	cambodia*.mp.	7156

111	indonesia*.mp.	30680

112	lao*.mp.	9346

113	burmese*.mp.	1246

114	filipino*.mp.	4797

115	vietnam*.mp.	31425

116	singapor*.mp.	27689

117	thai*.mp.	58438

118	Southeast Asian People/	1280

119	autosome disorder*.mp.	0

120	sonograph*, Prenatal.mp.	48

121	prenatal sonograph*.mp.	1319

122	fetal sonograph*.mp.	262

123	foetal ultrasound*.mp.	60

124	foetal ultrasonograph*.mp.	29

125	foetal sonograph*.mp.	13

126	malay*.mp.	41152

127	foetal echograph*.mp.	5

128	autosomal disorder*.mp.	257

129	1 or 2 or 3 or 4 or 5 or 6 or 7 or 8 or 9 or 10 or 11 or 12 or 13 or 14 or 15 or 16 or 17 or 18 or 19 or 20 or 21 or 22 or 23 or 24 or 25 or 26 or 27 or 28 or 29 or 30 or 31 or 32 or 33 or 34 or 35 or 36 or 37 or 119 or 128	159620

130	38 or 39 or 40 or 41 or 42 or 43 or 44 or 45 or 46 or 47 or 48 or 49 or 50 or 51 or 52 or 53 or 54 or 55 or 56 or 57 or 58 or 59 or 60 or 61 or 62 or 63 or 64 or 65 or 66 or 67 or 68 or 69 or 70 or 71 or 72 or 73 or 74 or 75 or 76 or 77 or 78 or 79 or 80 or 81 or 82 or 120 or 121 or 122 or 123 or 124 or 125 or 127	95431

131	83 or 84 or 85 or 86 or 87 or 88 or 89 or 90 or 91 or 92 or 93 or 94 or 95 or 96 or 97 or 98 or 99 or 100 or 101 or 102 or 103 or 104 or 105 or 106 or 107 or 108 or 109 or 110 or 111 or 112 or 113 or 114 or 115 or 116 or 117 or 118 or 126	349485

132	129 and 130 and 131	124

133	limit 132 to (yr="2010 -Current" and (english or thai))	71

**2. Web-search**

2.1 **Via each country Ministry of Health and Obstetrics and Gynaecology Website**
- Brunei
  - Ministry of Health Brunei Darussalam (<https://www.moh.gov.bn/>)
- Cambodia
  - Ministry of Health, Cambodia (<https://moh.gov.kh/>)
  - Société Cambodgienne de gynécologie et d’Obstétrique (SCGO) ([www.scgo-kh.com/](http://www.scgo-kh.com/))
- Indonesia
  - HIMSS (Healthcare Information and Management Systems Society) (<https://www.himss.org/sponsor-ministry-health-republic-indonesia>)
  - [Digital Transformation Office MoH Republic Indonesia](https://dto.kemkes.go.id/) (<https://www.kemkes.go.id/>)
  - Indonesian Society of Obstetrics & Gynecology ([www.pogi.or.id](http://www.pogi.or.id/))
- Laos
  - Ministry of Health, Laos (<https://moh.gov.la/>)
  - Laos Association of Obstetrics and Gynecology (via contact emails: alongkonepsvobgyn@gmail.com, [laosobgyn@gmail.com](mailto:laosobgyn@gmail.com))
- Malaysia
  - Ministry of Health, Malaysia (<https://www.moh.gov.my/>)
  - Obstetrical and Gynaecological Society of Malaysia (OGSM) ([www.ogsm.org.my](http://www.ogsm.org.my/))
- Myanmar
  - Ministry of Health, Myanmar (<https://www.mohs.gov.mm/>)
  - Obstetrical & Gynaecological Society Myanmar Medical Association ([www.myanmarogsociety.org](http://www.myanmarogsociety.org/))
- Philippines
  - Republic of the Philippines, Department of Health (<https://doh.gov.ph/>)
  - Philippine Obstetrical & Gynaecological Society, (Foundation) Inc. (<https://pogsinc.org/>)
- Singapore
  - Ministry of Health, Singapore (<https://www.moh.gov.sg/>)
  - Obstetrical & Gynaecological Society of Singapore ([www.ogss.net](http://www.ogss.net/))
- Thailand
  - Ministry of Public Health, Thailand (<https://www.moph.go.th/>)
  - The Royal Thai College of Obstetricians & Gynaecologists ([www.rtcog.or.th](http://www.rtcog.or.th/))
- Timor Leste
  - Ministry of Health (Timor-Leste) ([http://www.moh.gov.tl](http://www.moh.gov.tl/))
- Vietnam
  - Ministry of Health Vietnam (<https://moh.gov.vn/web/ministry-of-health>)
  - Vietnam Association of Gynecology & Obstetrics (contact via an email vago@gmail.com)
  2.2 **Google hand-searching:**

[“trisomy 21” OR “Down* Syndrome” OR “trisomy 18” OR “Edward* syndrome”  OR “trisomy 13” OR “Patau* syndrome”  OR “abnormal karyotyp*” OR “aneuploid*” OR “chromosome abnormal*” OR “chromosome disorder*”] AND “screening” AND [“prenatal” OR “antenatal” OR “antepartum” OR “fetal” OR “fetus” OR "foetal" OR "foetus"]
