# Supplementary material for: Prenatal Screening for Common Aneuploidy in Southeast Asian Countries: A Systematic Review of Challenges and Opportunities
Source: Prenat Diagn. 2026 May 23;46(9):1393–410. doi: 10.1002/pd.70171 (PMC13446533; doi:10.1002/pd.70171)
Supplement: Supplementary file 2 [file PD-46-1393-s003.docx]

**Appendix III: Studies conducted in Southeast Asian countries on the screening performance of prenatal aneuploidy screening**

| **Country (study)** | **Types of studies** | **Participant number** | **Sensitivity** | **Specificity** | **False positive** | **Positive predictive value** | **Negative predictive value** |
| --- | --- | --- | --- | --- | --- | --- | --- |
| **Nuchal translucency (NT) measurement** | | | | | | | |
| Thailand^78^ | Prospective | 7,820 singleton pregnancies:  T21: 20 (0.26%) | Cut-off point of ≥ 95^th^ percentile for foetal crown rump length | | | | |
|  |  |  | 55.0% (95%CI: 33.2–76.8%) for T21 | - | 4.9% (95%CI: 4.4–5.4%) for T21 | - | - |
| Thailand^79^ | Prospective | 7,126 singleton pregnancies:  T21: 11 (3.1%), T18: 7 (2%), T13: 5 (1.4%) | Cut-off point of ≥ 95^th^ percentile for foetal crown rump length | | | | |
|  |  |  | • T21: 61.1% (95%CI: 38.6–83.6%)  • T18: 41.2% (95%CI: 17.8–64.6%)  • T13: 45.5% (95%CI: 16.0–74.9%) | 95.1% (95%CI: 94.5–95.6%) for T21, T18 and T13 | 5% for T21 | - | - |
| Thailand^80^ | Prospective | 190 singleton or twin pregnancies:  T21: 4 (2.1%), T18: 4 (2.1%), T13: 3 (1.6%), 45, XO: 3 (1.6%), 47, XYY: 1 (0.5%), 47, XXY (0.5%) | Cut-off point of ≥ 95^th^ percentile for foetal crown rump length | | | | |
|  |  |  | 71.43% for all aneuploidies  • T21: 50%  • T18: 75%  • T13: 100% | 96.02% for all aneuploidies  • T21: 91.94%  • T18: 92.47%  • T13: 92.51% | 3.98% for all aneuploidies  • T21: 8.77%  • T18: 92.47%  • T13: 92.51% | 50.3% for all aneuploidies | - |
| Vietnam^92^ | Prospective | 2,500 singleton pregnancies:  T21: 16 (0.64%), T18: 7 (0.28%), 47,XXX: 2 (0.08%), 47, XXY: 4 (0.16%) | Cut-off point ≥ 3.0 mm | | | | |
|  |  |  | 17.2% for all aneuploidies  12.5% for T21 | 99.4% for all aneuploidies  99.1% for T21 | 0.6% for all aneuploidies  0.9% for T21 | 25.0% for all aneuploidies  8.33% for T21 | **-** |
|  |  |  | Cut-off point of ≥ 95^th^ percentile for foetal crown rump length | | | | |
|  |  |  | 58.6% for all aneuploidies  68.8% for T21 | 95.9% for all aneuploidies  95.4% for T21 | 4.1% for all aneuploidies  4.6% for T21 | 14.4% for all aneuploidies  8.37% for T21 | **-** |
|  |  |  | Cut-off point ≥ 2.4 mm (as proposed by the study for optimal accuracy in Vietnamese pregnant women) | | | | |
|  |  |  | 65.5% for all aneuploidies  75.0% for T21 | 95.7% for all aneuploidies  95.1% for T21 | 4.3% for all aneuploidies  4.9% for T21 | 15.2% for all aneuploidies  9.0% for T21 |  |
| **Nuchal translucency (NT) measurement, plus maternal age, plus foetal heart rate** | | | | | | | |
| Thailand^67^ | Retrospective | 8,473 singleton pregnancies:  T21: 28 (0.33%) | For T21 | | | | |
|  |  |  | Using the Thai algorithm (high-risk cut-off 1:250) | | | | |
|  |  |  | 78.6% (95%CI: 59.0-91.7%) | 83.4% (95%CI: 82.6-84.2%) | 16.6% | 1.5% (95%CI: 1.0-2.3%) | 99.9% (95%CI: 99.8-100%) |
|  |  |  | Using the Thai algorithm (high-risk cut-off 1:100) | | | | |
|  |  |  | 67.9% (95%CI: 47.6-84.1%) | 96.5% (95%CI:96.1-96.9%) | <5% | 6.1% (95%CI: 3.7-9.4%) | 99.9% (95%CI: 99.8-99.9%) |
|  |  |  | Using the Fetal Medicine Foundation (FMF) algorithm (high-risk cut-off 1:250) | | | | |
|  |  |  | 60.7% (95%CI: 40.6-78.5%) | 97.6% (97.3-98.0%) | 2.4% | 7.9% (95%CI: 4.7-12.3%) | 99.9% (95%CI: 99.8-99.9%) |
| Thailand^91^ | Retrospective | 8,491 singleton pregnancies  T21: 0.33%,  T18: 0.11%,  T13: 0.11% | Using the Fetal Medicine Foundation (FMF) algorithm (high-risk cut-off 1:250) | | | | |
|  |  |  | 52.2% (95%CI: 37.7-66.6%) for all aneuploidies  60.7% (95%CI: 42.6-78.8%) for T21 | 97.2% (95%CI: 96.9-97.6%) for all aneuploidies  97.6% (95%CI: 97.3-98.0%) for T21 | 2.8% for all aneuploidies  2.4% for T21 | 9.2% (95%CI: 5.7-12.7%) for all aneuploidies  7.7% (95%CI: 4.2-11.1%) for T21 | 99.7% (95%CI: 99.6-99.8%) for all aneuploidies  99.9% (95%CI: 99.8-99.9%) for T21 |
| **Nasal bone length (NBL) measurement** | | | | | | | |
| Singapore^76^ | Prospective | 9,882 singleton pregnancies:  T21: 22 (0.22%) | For T21 | | | | |
|  |  |  | 95.5% | - | 6.1% | 3.4% | - |
| Thailand^81^ | Prospective  (criteria: hypoplastic nasal bone (less than 5^th^ percentile for gestational age) | 351 singleton high-risk pregnancies:  T21: 11 (3.13%) | For T21 | | | | |
|  |  |  | 100% | 92% | 7.6% | 30% | 100% |
| Thailand^80^ | Prospective  (criteria: absent nasal bone) | 190 singleton or twin pregnancies:  T21: 4 (2.11%), T18: 4 (2.11%), T13: 3 (1.58%), 45, XO: 3 (1.58%), 47, XYY: 1 (0.53%), 47, XXY: 1 (0.53%) | 28.57% for all aneuploidies  • T21: 25%  • T18: 50%  • T13: 33.33% | 99.43% for all aneuploidies  • T21: 97.85%  • T18: 98.39%  • T13: 97.86% | 0.57% for all aneuploidies  • T21: 2.2%  • T18: 1.64%  • T13: 2.19% |  |  |
| Thailand^78^ | Prospective  (criteria: absent nasal bone) | 7,820 singleton pregnancies:  T21: 20 (0.26%) | For T21 | | | | |
|  |  |  | 20.0% (95%CI: 2.5–37.5%) | - | 2.2% (95%CI:1.8–2.5%) | - | - |
| **Second-trimester ultrasound** | | | | | | | |
| Thailand^89^ | Retrospective | 4,033 high-risk pregnancies:  T21: 43 (1.1%) | For T21 | | | | |
|  |  |  | 69.8% (95%CI: 56.0-83.5%) | 49.6% (95%CI: 48.1-51.2%) | 50.4% | - | - |
| **First-trimester serum analyte (Pregnancy-associated plasma protein A; PAPP-A plus human chorionic gonadotropin; hCG, without NT measurement)** | | | | | | | |
| Thailand^62^ | Retrospective  (high-risk cut-off 1:250) | 24,885 singleton pregnancies:  T21: 36 (0.14%) | Using the Thai-specific reference range | | | | |
|  |  |  | 77.78% (95%CI: 64.2–91.4%) | 92.51% (95%CI: 92.2–92.8%) | 7.5% | - | - |
|  |  |  | Using the Caucasian reference ranges (with racial factor correction) | | | | |
|  |  |  | 63.89% (95%CI: 48.2–79.6%) | 87.48% (95%CI: 87.1–87.9%) | 12.5% | - | - |
| Thailand^63^ | Prospective  (high-risk cut-off 1:250) | 29,692 singleton pregnancies:  T21: 53 (0.18%) | Using the Thai-specific reference range | | | | |
|  |  |  | 79.2% | - | 6.8% | - |  |
| Thailand^82^ | Retrospective  (high-risk cut-off 1:250) | 5,070 singleton pregnancies:  T21: 7 (0.14%), T13: 1 (0.02%) | Using the Caucasian reference ranges | | | | |
|  |  |  | 62.5% (95%CI: 25.9-89.8%) for all aneuploidies  • T21: 71.4% (95%CI: 30.3-94.9%) | 96.1% (95%CI: 95.5-96.6%) for all aneuploidies  • T21: 96.1% (95%CI: 95.6-96.7%)  • T18 and T13: 99.9% (95%CI: 99.8-99.9%) | • T21: 3.85%  • T18: 0.04%  • T13: 0.02% | 2.4% (95%CI: 0.91-5.9%) for all aneuploidies  • T21: 2.5% (95%CI: 0.92-6.1%) | 99.9 (95%CI: 99.8-99.9%) for all aneuploidies  • T21: 99.9% (95%CI: 99.8-99.9%)  • T18 100% (95%CI: 99.9-100%)  • T13: 99.9% (95%CI: 99.8-99.9%) |
| **Combined first-trimester screening (Pregnancy-associated plasma protein A; PAPP-A plus human chorionic gonadotropin; hCG, with NT measurement)** | | | | | | | |
| Indonesia, Thailand and Vietnam^75^ | Prospective  (high-risk cut-off 1:200) | 376 Indonesian singleton pregnancies, 564 Thai singleton pregnancies, 600 Vietnamese singleton pregnancies | For T21 | | | | |
|  |  |  | Using the Caucasian reference ranges with country-specific correction | | | | |
|  |  |  | Cannot be calculated as there were no T21 in this study | • Indonesia 93.77% (95%CI: 90.82–95.81%)  • Thailand 98.61% (95%CI: 97.16–99.32%)  • Vietnam 99.50% (95%CI: 98.54–99.83%) |  |  |  |
|  |  |  | Using the Caucasian reference ranges with overall Asian correction | | | | |
|  |  |  | Cannot be calculated as there were no T21 in this study | • Indonesia 92.68% (95%CI: 89.56–94.92%)  • Thailand 97.81% (95%CI: 96.13–98.77%)  • Vietnam 99.17% (95%CI: 98.06–99.64%) |  |  |  |
|  |  |  | Using the Caucasian reference range with no correction | | | | |
|  |  |  | Cannot be calculated as there were no T21 in this study | • Indonesia 94.04% (95%CI: 91.14–96.03%)  • Thailand 98.41% (95%CI: 96.89–99.19%)  • Vietnam 99.33% (95%CI: 98.30–99.74%) |  |  |  |
| Singapore^76^ | Prospective  (High-risk cut-off ≥ 1:300) | 12,585 singleton pregnancies | For T21 | | | | |
|  |  |  | 87.1% | 94.9% | 5.1% | 99.9% |  |
| Singapore^77^ | Retrospective  (high-risk cut-off 1:250) | 10,289 singleton pregnancies:  T21: 37 (0.36%), T18: 11 (0.11%), T13: 1 (0.01%) | For overall T21, 18, 13 | | | | |
|  |  |  | 87.76% (95%CI: 75.22–95.34%) | 97.61% (95%CI: 97.29–97.89%) | 2.39% | 14.93% (95%CI: 11.02–19.58%) | 99.94% (95%CI: 99.87–99.98%) |
| Thailand^66^ | Retrospective  (high-risk cut-off 1:250) | 3,928 singleton pregnancies:  T21: 4 (0.10%) | For T21 | | | | |
|  |  |  | 75% (95%CI: 19.41–99.37%) | 94.65% (95%CI: 93.69–95.33%) | - | 1.41% (95%CI: 0.79–2.49%) | 99.97% (95%CI: 99.85–100%) |
| **Triple test (Alpha-fetoprotein; AFP, human chorionic gonadotropin; hCG, unconjugated estriol; uE3)** | | | | | | | |
| Thailand^63^ | Prospective  (high-risk cut-off 1:250) | 12,232 singleton pregnancies:  T21: 21 (0.17%) | For T21 | | | | |
|  |  |  | Using the Caucasian reference ranges | | | | |
|  |  |  | 76.2% |  | 11% |  |  |
|  |  |  | Using the Thai reference ranges | | | | |
|  |  |  | 76.2% | - | 9.2% | - | - |
| Thailand^60^ | Prospective  (high-risk cut-off 1:250) | 20,229 singleton pregnancies:  T21: 35 (0.17%) | For T21 | | | | |
|  |  |  | Using the Caucasian reference ranges | | | | |
|  |  |  | 74.3% (95%CI: 59.8–88.8%) | 88.3% (95%CI: 87.9–88.7%) | 11.7% (95%CI: 11.2–12.1%) |  |  |
|  |  |  | Using the Thai reference ranges | | | | |
|  |  |  | 74.3% (95%CI: 59.8–88.8%) | 91.2% (95%CI: 90.8–91.6%) | 8.8% (95%CI: 8.4–9.2%) |  |  |
| **Quadruple test (Alpha-fetoprotein; AFP, human chorionic gonadotropin; hCG, unconjugated estriol; uE3, dimeric inhibin A; DI)** | | | | | | | |
| Thailand^61^ | Prospective  (high-risk cut-off 1:250) | 39,740 singleton pregnancies:  T21: 74 (0.19%) | For T21 | | | | |
|  |  |  | Using the Caucasian reference range | | | | |
|  |  |  | 81.1% (95%CI: 72.2-90.0%) | 92.8% (95%CI: 92.6-93.1%) | 7.2% (95%CI: 6.9-7.4%) |  |  |
|  |  |  | Using the Thai reference range | | | | |
|  |  |  | 87.8% (95%: 80.4-95.3%) | 93.2% (95%: 92.9-93.4%) | 6.8% (95%: 6.6-7.0%) |  |  |
| Thailand^83^ | Prospective  (high-risk cut-off 1:250) | 2,375 singleton pregnancies:  T21: 4 (0.17%) | For T21 | | | | |
|  |  |  | 75.0% | 91.4% | 8.6% | 100% |  |
| Thailand^64^ | Prospective  (high-risk cut-off 1:250) | 5,487 singleton pregnancies  T21: 12 (0.22%) | For T21 | | | | |
|  |  |  | Using the European reference range | | | | |
|  |  |  | 75.0% | 90.9% | 9.1% | 1.8% | 99.7% |
|  |  |  | Using the Thai reference range | | | | |
|  |  |  | 83.3% | 90.4% | 9.6% | 1.9% | 100% |
| Thailand^82^ | Retrospective  (high-risk cut-off 1:250) | 1,002 singleton pregnancies:  T21: 2 (0.20%) | Using Caucasian reference ranges | | | | |
|  |  |  | 50% (95%CI: 26.7-97.3%) for all aneuploidies  • T21: 50% (95%CI: 26.7-97.3%) | 93.5% (95%CI: 91.7-94.8%) for all aneuploidies  • T21: 93.9% (95%CI: 92.2-95.3%)  • T18: 99.6% (95%CI: 98.9-99.8%) | • T21: 6.1%  • T18: 0.4% | 1.5% (95%CI: 0.07-9.3%) for all aneuploidies  • T21: 1.6% (95%CI: 0.08-9.8%) | 99.9% (95%CI: 99.3-99.9%) for all aneuploidies  • T21: 99.9% (95%CI: 99.3-99.9%)  • T18: 100% (95%CI: 95.5-100%) |
| **Contingent screening (High risk: invasive diagnosis offered, intermediate risk: second-trimester screening needed, and low risk: no further test)** | | | | | | | |
| Thailand^65^ | Retrospective  (without NT measurement) | 24,874 singleton pregnancies:  T21: 36 (0.14%) | Using the upper cut-off: 1:30 and lower cut-off: 1:1500 | | | | |
|  |  |  | 88.9% | - | 8.5% | - | - |
|  |  |  | Using the upper cut-off: 1:50 and lower cut-off: 1:900 | | | | |
|  |  |  | 86.1% | - | 8.1% | - | - |
| Thailand^63^ | Prospective  (the upper cut-off: 1:30, lower cut-off: 1:1500) | 29,692 singleton pregnancies:  T21: 53 (0.18%) | Using the Caucasian reference range | | | | |
|  |  |  | 86.8% | - | 13.7% | - |  |
|  |  |  | Using the Thai reference range | | | | |
|  |  |  | 84.9% | - | 7.7% | - |  |
| **Non-invasive prenatal testing (NIPT) *(NGS = next‑generation sequencing, SNP = single‑nucleotide polymorphism)*** | | | | | | | |
| Thailand (NGS‑based)^88^ | Prospective | 571 pregnancies:  T21: 14 (2.5%), T18: 9 (1.6%), T13: 2 (0.4%), mosaic 45, XO: 2 (0.4%) | 100% concordance in all euploid/ aneuploid calls. | |  | • T21: 95.1%  • T18: 91.4%  • T13: 83.3%  • 45, XO: 74.2% |  |
| Thailand (NGS‑based)^84^ | Retrospective | 219 pregnancies:  T 21: 11 (5.0%), T18: 5 (8.2%), T13: 2 (0.9%) | • T21: 100%  • T18: 80%  • T13: 100% | • T21: 100%  • T18: 99.53%  • T13: 100% | - | • T21: 100%  • T18: 80%  • T13: 100% |  |
| Thailand (NGS‑based)^85^ | Prospective | 4,721 (singleton pregnancies - 96.47% and twin pregnancies - 3.53%):  T21: 30 (0.6%), T18: 12 (0.3%), T13: 4 (0.1%) | 100.00% (95%CI: 73.54, 100.00%) for all aneuploidies  • T21: 100.00% (95%CI: 88.43, 100%)  • T18: 100.00% (95%CI: 73.54, 100.00%)  • T13: 100.00% (95%CI: 39.76, 100.00%) | 99.87% (95%CI: 99.72, 99.95%) for all aneuploidies  • T21: 99.98% (95%CI: 99.88, 100.00%)  • T18: 99.92% (95%CI: 99.78, 99.98%)  • T13: 99.98% (95%CI: 99.88, 100.00%) | - | 88.46% (95%CI: 76.56, 95.65%) for all aneuploidies  • T21: 96.77% (95%CI: 83.30, 99.92%)  • T18: 75.00% (95%CI: 47.62, 92.73%)  • T13: 80.00% (95%CI: 28.36, 99.49%) |  |
| Thailand (NGS- based; paired-end sequencing)^86^ | Retrospective | 27 twin pregnancies: T21: 2 (7.41%), T18: 1 (3.70%) | For overall T21 and T18 | | | | |
|  |  |  | 100% | 100% | 0 | 100% | 100% |
| Thailand (SNP-based)^87^ | Retrospective | 8,434 singleton pregnancies:  T21:48 (0.57%), T18:15 (0.18%), T13: 7 (0.08%) | Incomplete post-test follow-up limits accurate estimation of sensitivity and specificity. | | •T21: 3 cases  • T18: 0 case  • T13: 5 cases | 89.6% (95%CI: 81.2–94.5) for overall T21, T18 and T13  • T21: 94% (95%CI: 83.5–98.0%)  • T18: 100%  • T13: 58.3% (95%CI: 36.8–77.1%) | Incomplete post-test follow-up limits accurate estimation of NPV. |
| Thailand^82^ | Retrospective | 1,778 singleton pregnancies:  T21: 16 (0.90%), T18: 6 (0.34%), T13: 1 (0.06%) | 100% (95%CI: 82.2-100%) for all aneuploidies  • T21: 100% (95%CI: 75.9-100%)  • T18: 100% (95%CI: 51.7-100%)  • T13: 100% (95%CI: 5.5-100%) | 100% (95%CI: 99.7-100%) for all aneuploidies,  each T21, T18 and T13 | 0 | 100% (95%CI: 82.2-100%) for all aneuploidies  • T21: 100% (95%CI: 75.9-100%)  • T18: 100% (95%CI: 51.7-100%)  • T13: 100% (95%CI: 5.5-100%) | 100% (95%CI: 99.7-100%) for all aneuploidies,  each T21, 18 and 13 |
| Thailand (NGS- based)^90^ | Retrospective | 19,704 pregnancies:  T21: 48 (0.24%), T18: 17 (0.09%), T13: 7 (0.04%) | • T21: 97.92% (95%CI: 88.93–99.95%)  • T18: 94.12% (95%CI: 71.31–99.85%)  • T13: 85.71% (95%CI: 42.13–99.64%) | • T21: 99.96% (95%CI: 99.93–99.99%)  • T18: 99.96% (95%CI: 99.93–99.99%)  • T13: 99.97% (95%CI: 99.93–99.99%) | • T21: 7 cases  • T18: 7 cases  • T13: 6 cases | • T21: 87.04% (95%CI: 75.10–94.63%)  • T18: 69.57% (95%CI: 47.08–86.79%)  • T13: 50% (95%CI: 21.09–78.91%) | • T21: 99.99% (95%CI: 99.97–100%)  • T18: 99.99% (95%CI: 99.97–100%)  • T13: 99.99% (95%CI: 99.97–100%) |
| Vietnam^93^ | Retrospective | 6,048 singleton pregnancies:  T21: 8 (0.13%), T18: 2 (0.03%), T13: 5 (0.08%) | 100% (for each T21, T18 and T13) | • T21: 99.98%  • T18: 99.97%  • T13: 99.95% | • T21: 1 case  • T18: 2 cases  • T13: 3 cases | • T21: 88.89%  • T18: 50.00%  • T13: 62.50% | 100% for each T21, T18 and T13 |
| Vietnam^94^ | Retrospective (September 2018 to August 2019) and prospective (September 2019 to August 2020) | 208 high-risk pregnancies: T21, 18, 13: 8 (3.85%) | For overall T21, 18 and 13 | | | | |
|  |  |  | 100% (95% CI: 63 – 100%) | 99.5% (95% CI: 97.2 – 99.9%) | - | 21.9% (95% CI: 3.8 – 66.4%) | - |
| Vietnam (NGS‑based; triSure algorithm)^17^ | Prospective | 130 high-risk singleton pregnancies:  T21: 43 (33.1%), T18: 11 (8.5%), T13: 4 (3.1%) | 98.3% for overall T21, T18 and T13  T21: 97.7%  T18: 100%  T13: 100% | 98.6% for overall T21, T18 and T13  T21: 98.9%  T18: 100%  T13: 100% | - | - | - |
